# Is vegetarian diet associated with a lower risk of breast cancer in Taiwanese women?

**DOI:** 10.1186/s12889-017-4819-1

**Published:** 2017-10-10

**Authors:** Yao-Jen Chang, Yi-Cheng Hou, Li-Ju Chen, Jing-Hui Wu, Chao-Chuan Wu, Yun-Jau Chang, Kuo-Piao Chung

**Affiliations:** 1 School of Medicine, Buddhist Tzu Chi University, Hualien, Taiwan; 2 Department of Surgery, Taipei Tzu Chi Hospital, Buddhist Tzu Chi Medical Foundation, New Taipei City, Taiwan; 3 Division of Nutrition, Taipei Tzu Chi Hospital, Buddhist Tzu Chi Medical Foundation, New Taipei City, Taiwan; 4 Graduate Institute of Health Policy and Management, College of Public Health, National Taiwan University, Taipei, Taiwan; 5 Department of Ophthalmology, Heping Branch, Taipei City Hospital, Taipei, Taiwan; 6 Department of General Surgery, Zhongxing Branch, Taipei City Hospital, No.145, Zhengzhou Rd., Datong District, Taipei, Taiwan; 7 Department of General Surgery, National Taiwan University Hospital, Taipei, Taiwan

**Keywords:** Breast cancer risk, Dietary pattern, Vegetarian diet, Isoflavones

## Abstract

**Background:**

Studies on the relationship between vegetarian diet and breast cancer in Asian populations are limited. This study aimed to investigate the relationship between vegetarian diet, dietary patterns, and breast cancer in Taiwanese women.

**Methods:**

This case-control study compared the dietary patterns of 233 breast cancer patients and 236 age-matched controls. A questionnaire about vegetarian diets and 28 frequently-consumed food items was administered to these 469 patients in the surgical department of Taipei Tzu Chi Hospital. Serum biochemical status was also examined.

**Results:**

There were no significant differences between the two groups for age, education, family history, oral contraceptive usage, or regular exercise. However, the cancer group presented with both a higher body mass index and an older age of primiparity (*P* < 0.05). Two food items (shellfish and seafood) were highly correlated (correlation coefficient = 0.77), so shellfish was excluded to avoid multicollinearity. A factor analysis of 27 food items produced five dietary patterns: meat, processed meat, fruit/vegetable/soybean, dessert/sugar, and fermented food. Multivariate logistic regression showed that meat/fat and processed meat dietary patterns were associated with breast cancer risk (odds ratio (OR): 2.22, 95% CI 1.67–2.94, *P* < 0.001; OR: 1.49, 95% CI 1.09–2.04, *P* = 0.013, respectively). Vegetarian diet, high isoflavone intake, and high albumin levels were inversely associated with breast cancer risk (*P* < 0.05). Vegetarians had a higher daily soy isoflavone intake than non-vegetarians (25.9 ± 25.6 mg vs. 18.1 ± 15.6 mg, *P* < 0.001).

**Conclusions:**

Vegetarian diets show as protective role against breast cancer risk, while meat and processed meat dietary patterns are associated with a higher breast cancer risk.

**Electronic supplementary material:**

The online version of this article (10.1186/s12889-017-4819-1) contains supplementary material, which is available to authorized users.

## Background

In Taiwan, breast cancer is the most common type of cancer in females and the fourth leading cause of female cancer deaths since 2003 [1]. The incidence of breast cancer has doubled in just two decades. Additionally, breast cancer has been shown to be the fastest increasing cancer type, according to one administration’s report [2]. Literature has suggested varying risk factors for the development of breast cancer in premenopausal and postmenopausal women, including genetic factors and environmental factors [3, 4]. Among these risk factors, diet has attracted considerable attention because it is a modifiable risk factor and thus offers opportunities to design preventive strategies [5]. For decades, researchers have considered diet to be the most important of the environmental factors [6, 7]. Some scholars have reported that the influence of diet is more important in breast cancer development than that of genetics [8].

The role that nutrients and food components play in the development of breast cancer has been comprehensively examined. Caffeinated coffee intake has been shown to likely be associated with a lower risk of postmenopausal breast cancer [9]. High dietary fat and sugar intake may increase breast cancer risk [10, 11] while poultry, vegetable, and fruit consumption may decrease breast cancer risk [12–14]. Despite these findings, other studies have presented disagreeing or incongruent results about the risk factors of breast cancer [15, 16]. Additionally, varying conclusions from studies examining individual nutrients or food components as risk factors for the breast cancer have prevented researchers from proposing adequate dietary recommendations for decreasing cancer incidence [17]. As people do not exclusively consume individual foods, but rather combinations of them, an assessment of dietary patterns might offer valuable information when determining the association between diet and cancer risk [18]. Therefore, examining dietary patterns to assess risk factors is a reasonable approach, which researchers have used to investigate the association of food and the risk of many cancers including: esophageal cancer [19], gastric cancer [20], colorectal cancer [21], pancreatic cancer [22], hepatocellular carcinoma [23], and breast cancer [24]. In Asian countries, such as Taiwan, vegetarianism prevails due to religious beliefs, creating a unique dietary pattern in these regions. One western study showed that a vegetarian diet may decrease the risk of cancers such as colorectal cancer [25]. However, there is still limited evidence regarding the association between vegetarianism and breast cancer in Asian populations. This study had two main objectives. First, we sought to investigate whether dietary patterns might be a risk factor for breast cancer. Second, we sought to examine the association between vegetarianism and breast cancer.

## Methods

### Study design

This case-control study was conducted at the Taipei Tzu Chi Hospital from April, 2010 to December, 2013. The admission of patients into the study followed a protocol similar to that used in the preliminary report of this study [26]. The research protocol was approved by the institutional review board at the Taipei Tzu Chi Hospital (01-XD22–047). Signed consent was obtained from all participants.

### Study cohort

Inclusion criterion for the cancer group was female patients (≤85 years of age) who were diagnosed with primary breast cancer. Research team members contacted potential participants to introduce the study and obtained their signed informed consent. Among 250 eligible women, 17 patients (6.8%) declined, and 233 patients (93.2%) were interviewed. Recruitment of the control group was based on individuals who underwent health examinations for breast lesions during the same time period at the same hospital. Inclusion criteria for the control group consisted of the following: no history of breast cancer, residence in the same neighborhood, and age within a 5-year margin of the cancer group. Individuals who were on controlled diets for diabetes or other diseases were excluded. As performed in the cancer group, research team members contacted potential control group candidates and scheduled interviews for those who agreed to participate. Among women eligible to participate in the control group (500 contacted), 274 declined to answer questionnaires (mostly due to time limitations or factors unrelated to dietary pattern), and 236 (47.2%) agreed and completed questionnaires. Questionnaires were administered at the study hospital.

### Dietary intake

The first section of the questionnaire contained demographic information including body mass index (BMI). The second section contained a Food-Frequency Questionnaire (FFQ) with 28 separate food items. Daily dietary intake of each participant was assessed by the FFQ, which was developed and used for the third Taiwan National Nutrition and Health Survey conducted from 1993 and 1996 [26, 27]. For each food item, participants were asked how frequently (e.g. 1 time per day, 4 times per week or 9 times per month) they consumed each food during recent month. For example, a food consumed only once per month would result in a monthly score of 0.03 (1/30). The third section of the questionnaire contained 4 questions about vegetarian dietary patterns, 2 questions about vitamins, minerals and other supplements, and 2 questions about tobacco and alcohol consumption. Vegetarian diet referred to ovo-vegetarian, lacto-vegetarian, lacto-ovo vegetarian or vegan diet which excluded all animal products including eggs and dairy products. For women in the breast cancer group, the questionnaire was administered the day before their scheduled surgery, while in control group the questionnaire was administered before each participant’s clinic appointment. The amount of daily dietary soy isoflavone intake was calculated according to a previously described method [28, 29]. Blood samples were also drawn to determine levels of albumin, triglyceride, and estradiol.

### Statistical analyses

Mean values (with standard deviations) and frequencies were reported for the demographic and dietary characteristics of each group. Independent-sample t tests were used to compare continuous variables. Chi-square tests were used to compare categorical variables. Bivariate correlation analyses across food items were conducted to avoid multicollinearity. We used factor analysis according to monthly FFQ scores to derive dietary patterns for subsequent inferential statistics. Researchers frequently use factor analysis to derive dietary patterns [30–32]. Theoretically, factor analysis is based on the correlation matrix of observed variables, which allows the extraction unobserved factors (latent variables) either by non-rotated or rotated methods (e.g., orthogonal, oblique rotation). The coefficients, called factor loadings, are the correlations between each food item and each factor. Factor scores were calculated by multiplying the factor loadings (varimax in this study) with the corresponding standardized value for each food and summing across the food items. For each participant, the factor scores indicated the extent to which the diet aligned with the respective dietary patterns. A high factor score for a given pattern indicated frequent intake of the foods within that food pattern, and a low score indicated low intake of those foods. The numbers of factors extracted were based on the following criteria: eigenvalue >1, scree plot, total percent variance explained and meaningful factors [33].

Relative risks computed from logistic regression were reported as odds ratios (ORs) and 95% confidence intervals (CIs). All analyses were conducted using SPSS (Statistical Package for the Social Science 18.0). Statistical tests were two-sided. In all tests, a *P* < 0.05 was considered statistically significant.

## Results

The demographic characteristics of participants are shown in Table 1. There were no significant differences in age, education level, menarche, oral contraceptive use, hormone replacement therapy, or regular exercise between groups. The cancer group had a higher mean BMI (24.32 ± 3.92 vs. 22.98 ± 3.42, *P* < 0.001), higher percentage of primiparity >30 years (*P* = 0.011), and lower exercise frequency (*P* = 0.009) than the control group. The cancer group also had a lower percentage of women following vegetarian diets (34.3% vs. 48.7%, *P* = 0.002) and lower daily soy isoflavone intake (17.2 ± 16.0 mg vs. 26.3 ± 24.7 mg, *P* < 0.001) than the control group. Additionally, serological tests showed that participants in the cancer group had lower serum albumin levels (3.85 ± 0.36 vs. 4.12 ± 0.27, *P* = 0.004). There was no difference in serum estradiol levels between the breast cancer and control groups (*P* = 0.205). Monthly food frequency was the highest for tea (5.0; see Additional file 1: Table S1), followed by fish, poultry, livestock, soybeans, milk, eggs, vegetables, mushrooms, fruit, coffee, sweets, and beverages (3.0). Monthly food frequency was the lowest for sashimi (0.29). In the cancer group, monthly food frequencies of fish, poultry, livestock, streaked meat, smoked meat, processed meat (sausage), shellfish, seafood, milk (whole fat), sweets and sugar intake were significantly different than those of the control group.

**Table 1:** Demographic characteristics and laboratory results of participants

	Total (*N* = 469)		Breast cancer (*N* = 233)		Control (*N* = 236)		
Variable	Range	Mean ± SD	Range	Mean ± SD	Range	Mean ± SD	*P* value
Age (years)	28–83	55.4 ± 9.7	28–83	55.9 ± 10.4	29–80	54.9 ± 9	0.252
BMI (kg/m^2^)	15.98–38.34	23.65 ± 3.73	16.21–38.34	24.32 ± 3.92	15.98–36.51	22.98 ± 3.42	<0.001
Education							0.153
Junior high school or lower		198 (42.2)		106 (45.5)		92 (39.0)	
Senior high school or higher		271 (57.8)		127 (54.5)		144 (61.0)	
Menarche							0.117
11–14 years old		305 (65.0)		160 (68.7)		145 (61.4)	
15 years old		89 (19.0)		35 (15.0)		54 (22.9)	
≥ 16 years old		75 (16.0)		38 (16.3)		37 (15.7)	
Primiparity							0.011
≤ 30 years		348 (74.2)		160 (68.7)		188 (79.7)	
> 30 years		59 (12.6)		39 (16.7)		20 (8.5)	
never		62 (13.2)		34 (14.6)		28 (11.9)	
Menopause							<0.001
Not yet		135 (28.9)		79 (34.1)		56 (23.7)	
< 45 years old		71 (15.2)		32 (13.8)		39 (16.5)	
45–55 years old		247 (52.8)		111 (47.8)		136 (57.6)	
≥ 55 years old		15 (3.2)		10 (4.3)		5 (2.1)	
Family history (BC)							0.390
Present		41 (8.7)		23 (9.9)		18 (7.6)	
Absent		428 (91.3)		210 (90.1)		218 (92.4)	
Oral contraceptive							0.604
Current user		18 (3.8)		7 (3.0)		11 (4.7)	
Prior user		97 (20.7)		47 (20.2)		50 (21.2)	
Never		354 (75.5)		179 (76.8)		175 (74.1)	
Hormone replacement therapy							0.503
Never		386 (82.3)		189 (81.1)		197 (83.5)	
Ever		83 (17.7)		44 (18.8)		39 (16.5)	
Regular exercise							0.358
Yes		191 (40.7)		90 (38.6)		101 (42.8)	
No		278 (59.3)		143 (61.4)		135 (57.2)	
Exercise frequency							0.009
0		275 (58.6)		142 (60.9)		133 (56.4)	
0.5–4/week		115 (24.5)		44 (18.9)		71 (30.1)	
> 4.1/week		79 (16.8)		47 (20.2)		32 (13.6)	
Exercise time							0.884
≤ 30 min		363 (77.4)		181 (77.7)		182 (77.1)	
> 30 min		106 (22.6)		52 (23.3)		54 (22.0)	
Vegetarian (≥12 months)							0.002
yes		195 (41.6)		80 (34.3)		115 (48.7)	
no		274 (58.4)		153 (65.7)		121 (51.3)	
Daily isoflavones intake^a^	0–285	21.8 ± 21.3	0–88.5	17.2 ± 16	0–285	26.3 ± 24.7	<0.001
Serum level							
Albumin level (g/dL)	2.4–5.0	3.99 ± 0.34	2.4–4.8	3.85 ± 0.36	3.1–5.0	4.12 ± 0.27	0.004
Triglyceride (mg/dL)	21–681	106.1 ± 62.8	21–438	107.5 ± 62.6	25–681	104.7 ± 63.1	0.628
Estradiol (pg/ml)	2.5–787	55.8 ± 92.6	2.5–693	50.3 ± 74.6	5.2–786.4	61.2 ± 107.4	0.205

*BMI* body mass index (=BW/BH^2^, *BW* body weight; *BH* body height); *SD* standard deviation; ^a^ mg of isoflavones (aglucon equivalents) / 100 g of wet weight

**Fig. 1:**
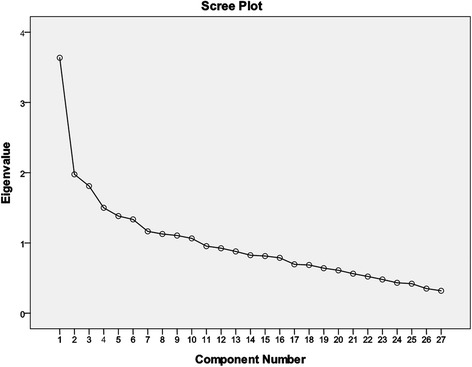
Scree plot of factor analysis

Correlations of 28 food items showed a high correlation (correlation coefficient = 0.77) between seafood (shrimp, crab) and shellfish (oyster, clam). Therefore, shellfish was excluded from subsequent analyses, which computerized only twenty-seven food items. Figure 1 shows the scree plot from exploratory factor analysis (EFA). Five dietary patterns (meat, processed-meat, fruit/vegetables/soybean, dessert/sugar, and fermented foods) with eigenvalues >1.5 were extracted according to the scree plot and factor loading matrix after orthogonal (varimax) rotation. Together, these five patterns accounted for 38.2% of the total variation in the analysis. Table 2 summarizes the factor loadings (>0.2) for the food items in each dietary pattern in this study (See Additional file 1: Table S2 for all non-rotated factor loadings; see Additional file 1: Table S3 for all rotated factor loadings). Univariate analysis revealed that the meat and processed-meat dietary patterns were associated with breast cancer risk, while others were not. Multivariate analysis also showed that meat and processed-meat patterns were significantly associated with breast cancer risk (OR 2.25, 95% CI: 1.69–2.98, *P* < 0.001; OR 1.56, 95% CI: 1.14–2.13, *P* = 0.006, respectively, see Table 3). Within the cancer group, comparing the dietary oil consumption of patients in the fourth and first quartiles within the meat/fat dietary pattern indicated that the women in the fourth quartile consumed both more meat and more dietary oil (Additional file 1: Table S4) than those in the first.

**Table 2:** Factor loadings for the six major dietary patterns among participants

	Dietary pattern				
	Animal source		Plant source		
Food item^a^	Meat	Processed meat	Fruit/Vegetables/Soybean	Dessert/sugar	Fermentation
Poultry (chicken, duck, goose)	0.744				
Fish (sea fish, fresh water fish)	0.741				
Livestock (pork, beef, lamb)	0.688			0.230	
Seafood (shrimp, crab, squid, fish viscera, spawn)	0.594	0.327			0.257
Streaked meat (fatty meat)	0.519				
Sashimi (sliced fresh fish meat)	0.464			0.308	
Smoked meat (smoked chicken, smoked pork, smoked sausage, smoked bacon, smoked hot dog)		0.664			
Fried food		0.604		0.261	
Internal organ (liver, heart, kidney, colon, intestine)	0.433	0.564			0.313
Processed meat (sausage, bacon, cured meat, ham, pork floss)	0.321	0.559			0.262
Soybeans (dried tofu)			0.731		
Soybeans (soybean milk)			0.639		
Mushrooms			0.647		
Fruit	0.207	−0.246	0.531		
Vegetables		−0.305	0.392	−0.248	
Sugar (e.g., candy)				0.630	0.234
Sweets (e.g., cake, sweet bun)				0.614	
Beverage		0.373		0.469	
Fermented food (e.g., miso)					0.767
Pickled food (e.g., kimchi)					0.703
Eggs	0.234	0.274	0.220	0.247	
Coffee				0.273	
Milk (low fat)		−0.228			
Milk (skim fat)				−0.221	
Eigenvalue	3.010	2.161	1.996	1.607	1.531
Variance explained (%)	11.15	8.01	7.39	5.95	5.67

^a^ values of factor loading <0.200 (absolute value) are not shown; fresh fruit juice, milk (whole fat), and tea have not been listed due to factor loading <0.200

**Table 3:** Dietary patterns and breast cancer risk

	Breast cancer					
	Univariate analysis			Multivariate analysis^a^		
Dietary pattern	OR	95% CI	*P* value	OR	95% CI	*P* value
Meat (nil)			<0.001			<0.001
1 time/day	1.85	(1.47–2.33)		2.25	(1.69–2.98)	
Processed meat (nil)			0.052			0.006
1 time/day	1.22	(1.00–1.49)		1.56	(1.14–2.13)	
Fruit/Vegetables/Soybean (nil)			0.834			0.909
1 time/day	0.98	(0.83–1.25)		1.01	(0.82–1.26)	
Dessert/sugar (nil)			0.490			0.403
1 time/day	0.93	(0.78–1.12)		0.90	(0.71–1.14)	
Fermented food (nil)			0.803			0.850
1 time/day	0.98	(0.81–1.17)		0.98	(0.76–1.25)	

Characters in parentheses indicate reference group; ^a^: estimates of logistic regression were adjusted for age, body mass index, albumin, triglyceride, total cholesterol, estradiol, smoke, alcohol, family history of breast cancer; OR = odds ratio, CI = confidence interval

Table 4 summarizes other significant factors for breast cancer including a vegetarian diet (OR 0.42, 95% CI: 0.27–0.65, *P* < 0.001), BMI > 23 kg/m^2^ (OR 2.00, 95% CI: 1.28–3.13, *P* = 0.003), primiparity >30 years (OR 2.38, 95% CI: 1.43–4.00, *P* = 0.001), serum albumin > 4 g/dl (OR 0.30, 95% CI: 0.19–0.47, *P* < 0.001), and daily soy isoflavone intake > 22 mg (OR 0.37, 95% CI: 0.24–0.60, *P* < 0.001). Serum triglyceride and estradiol levels were not associated with breast cancer risk (*P* > 0.05). Vegetarian and non-vegetarian participants had similar BMI (23.4 vs. 23.8, *P* = 0.262). Vegetarian participants consumed higher daily quantities of soy isoflavones than non-vegetarian participants (25.9 ± 25.6 vs. 18.1 ± 15.6, *P* < 0.001, Table 5), but their serum triglyceride and estradiol levels were not different than those of non-vegetarian participants.

**Table 4:** Risk factor for breast cancer

	Breast cancer		
Variable	OR^a^	95% CI	*P* value
Vegetarian diet			
< 12 months	1	–	–
≥ 12 months	0.42	(0.27–0.65)	<0.001
Tobacco			
No	1	–	–
Yes	1.41	(0.68–2.86)	0.364
Alcohol			
No	1	–	–
Yes	0.92	(0.49–1.72)	0.794
Family history			
No	1	–	–
Yes	0.76	(0.40–1.45)	0.399
BMI			
≤ 23 kg/m^2^	1	–	–
> 23 kg/m^2^	2.00	(1.28–3.13)	0.003
Primiparity			
≤ 30 years	1	–	–
> 30 years	2.38	(1.43–4.00)	0.001
Albumin			
≤ 4 g/dl	1	–	–
> 4 g/dl	0.30	(0.19–0.47)	<0.001
Soy isoflavones intake			
≤ 22 mg	1	–	–
> 22 mg	0.37	(0.24–0.60)	<0.001
Triglyceride			
≤ 106 mg/dl	1	–	–
> 106 mg/dl	0.90	(0.56–1.46)	0.684
Estradiol			
≤ 56 pg/ml	1	–	–
> 56 pg/ml	0.86	(0.46–1.61)	0.639

^a^: estimates of logistic regression were adjusted for age, regular exercise; OR = odds ratio, CI = confidence interval; characters in parentheses indicate reference group

**Table 5:** Comparison of soy isoflavones intake and blood tests between vegetarian and non-vegetarian

	Vegetarian (*N* = 223)	Non-vegetarian (*N* = 246)	
Variable	Mean ± SD	Mean ± SD	*P* value
Soy isoflavones (mg)^a^	25.9 ± 25.6	18.1 ± 15.6	<0.001
Serum level			
Albumin level (g/dl)	4.01 ± 0.35	3.97 ± 0.33	0.151
Triglyceride (mg/dl)	109.3 ± 73.0	103.2 ± 51.9	0.291
Estradiol (pg/ml)	50.3 ± 86.4	60.8 ± 97.9	0.219

^a^ mg of isoflavones (aglucon equivalents)/ 100 g of wet weight

## Discussion

Our study results show that dietary patterns are associated with breast cancer risk in Taiwanese women and support a protective role of vegetarian diets against developing breast cancer. Two out of five dietary patterns (meat and processed-meat) derived from factor analysis were significantly associated with breast cancer risk using both univariate and multivariate analyses. Other dietary patterns (fruit/vegetable/soybean, dessert/sugar, and fermented foods) were not associated with breast cancer risk. Our results are partially consistent with a prior study in Taiwanese women, which showed a harmful effect of dietary fat on the risk of breast cancer. However, that same study did not observe the protective effect of soy dietary patterns found in this study [34]. In studying Caucasian women, Chandran et al. also found that the consumption of processed and/or unprocessed red meat and poultry increased breast cancer risk [12]. Therefore, our results are consistent with prior literature in suggesting that foods from animal sources, rather than plant sources, may contribute to the development of breast cancer.

However, there are some disagreements between our results and those from other studies. Zhang et al. reported a diet characterized by the high consumption of vegetables, fruit, soy, milk, poultry and fish was associated with a lower risk of breast cancer in Chinese women, while refined grains, red meat and pickled foods were associated with higher risk of breast cancer [35]. A study by Cho et al. in Korean women observed that a diet rich in vegetables and seafood was associated with decreased breast cancer risk [36]. In the United Kingdom, it was shown that a fish-eating dietary pattern that excludes other meats might reduce breast cancer risk [37]. Finally, in German women, an unhealthy dietary pattern (high meat and deep-fried fat intake) was not associated with higher risk of breast cancer than a healthy dietary pattern (high vegetable and vegetable oil intake) [38].

The term “vegetarian diet” refers to a special dietary pattern that precludes meat-fat and processed meat dietary patterns. Several recent studies have reported that plant-based dietary patterns are associated with a reduced breast cancer risk [24, 39]. Is being a vegetarian associated with a reduced risk of breast cancer? A high percentage of vegetarian (41.6%) among the patients of our Buddhist hospital makes it a unique and ideal place to investigate the impact of keeping a vegetarian diet on breast cancer risk. Our observations demonstrated that three non-meat dietary patterns had no association with breast cancer risk. As expected, vegetarians (of at least 1-year duration) had a lower odds ratio (0.42) for developing breast cancer than non-vegetarians. This finding was consistent with a prior study that demonstrated that lifelong meat abstention such as that typically found in South Asia might be protective against breast cancer [40]. Another large study in the USA also suggested, among subtypes of vegetarians, that a vegan diet might confer lower risk for female-specific cancers including breast cancer [18]. In women of the United Kingdom, it was reported that the vegetarian group had a 12% reduction in breast cancer risk, but the finding was not statistically significant [37].

The reasons explaining the reduction in breast cancer risk for vegetarians remain unclear. Reasonable explanations might be that vegetarian and vegan diets increase the consumption of beneficial plant foods and plant constituents, eliminate the intake of red and processed meat, and aid in achieving and maintaining an ideal weight [41]. Because of their high protein content, vegetarians in Asia consume soybean or soy products, which are more versatile than Western vegetarians [42]. Literature concerning hematological and biochemical comparisons of vegetarians and non-vegetarians is limited. One study found that vegans have lower BMIs than non-vegetarians but no differences were found in their functional immunocompetence [43]. Another study showed that there were no significant differences in testosterone or estradiol levels between vegetarians and non-vegetarians [44]. Our study had similar results. In 21,856 Japanese women, Yamamoto et al. reported that frequent miso soup and isoflavone consumption was associated with a reduced risk of breast cancer [45]. Our study found that the average daily soy isoflavone intake was higher for vegetarians than non-vegetarians (Table 5). Additionally, higher isoflavone intake was associated with a lower risk of breast cancer (OR = 0.37, Table 4), which suggests a potential protective role of isoflavones against breast cancer. Contrastingly, the finding that soy isoflavone intake is significantly associated with a reduced risk of breast cancer in Asian populations was not observed in Western populations by Dong et al. [46].

Isoflavones, a class of phytoestrogens, are found predominantly in soybeans and soy products [47]. The major constituents of isoflavones, daidzein and genistein, are known to interact with the alpha- and beta-estrogen receptors [48]. They may act as mimics of estrogen, thus regulating estrogen levels. This regulation can happen either by isoflavones acting as a weak estrogen when body estrogen levels are low or by inhibiting estrogen’s effects when body estrogen levels were high [49]. As vegetarians and vegans are typically frequent soy consumers, serum isoflavone levels may increase dramatically in these groups [50]. In our study, we noted the mean isoflavone level of non-vegetarians (18.1 mg) was within a 1 mg margin of that of the cancer group (17.2 mg). Furthermore, the mean isoflavone level of vegetarians (25.9 mg) was within a 1 mg margin of control group (26.3 mg). Therefore, in addition to the finding that vegetarians had lower breast cancer risk, our results support a possible chemopreventive effect of isoflavones.

Researchers have investigated the association between dietary patterns and breast cancer using various definitions. In Chinese women, Cui et al. observed that a meat/sweet pattern was associated with breast cancer risk, while a vegetable/soy pattern was not [31]. Wu et al. studied the dietary patterns of Asian-American breast cancer patients and found that women who consumed high amounts of Western meat/starch and ethnic-meat/starch had a higher risk of breast cancer [30]. Around the same time, Butler et al. reported that a vegetable-fruit-soy dietary pattern could lower the risk of breast cancer more than a meat-dim-sum dietary pattern in Chinese women in Singapore [32]. Cottet et al. reported an alcohol/Western dietary pattern was associated with a higher breast cancer risk for women in France [51]. Cade et al. found fish eaters had an inverse association with breast cancer in postmenopausal women in the United Kingdom [37]. More recently, Catsburg et al. observed that a meat/potatoes pattern was associated with an increased risk of breast cancer for Canadian women [24].

There are several limitations of this study. First, the study did not use a longitudinal study design. Therefore, the results cannot be used to infer a causal relationship between diet and breast cancer risk. Second, the number of participants is relatively small, which might limit our ability to differentiate the risks of women who were life-long vegetarians from those who became vegetarian only in later life. The extrapolation of results to the general population should be performed cautiously. Future studies may require more participants and a longer follow-up period. Finally, an inherent limitation originates from the factor analysis, as it captures the correlated profiles of variables that may or may not have any association with the disease. The dietary patterns derived from factor analysis reflect existing eating patterns of participants and may not necessarily be those patterns that are optimal for cancer prevention.

## Conclusions

Our findings demonstrate a significant positive correlation between breast cancer incidence and the meat and processed meat dietary patterns. They also demonstrate that vegetarian diet is associated with a reduced incidence of breast cancer. Higher BMI and older age of primiparity are also risk factors of breast cancer. In contrast, higher isoflavone intake and serum albumin levels are associated with a lower incidence of breast cancer. Vegetarian diets can increase isoflavone intake, which may partially contribute to the protective role of vegetarian diet against developing breast cancer. Large study samples are required to validate this association.

## Additional file

Additional file 1: Food frequency of participants and factor loadings for dietary patterns. (DOC 187 kb)
