# Supplementary material for: Is vegetarian diet associated with a lower risk of breast cancer in Taiwanese women?
Source: BMC Public Health. 2017 Oct 10;17:800. doi: 10.1186/s12889-017-4819-1 (PMC5635543; doi:10.1186/s12889-017-4819-1)
Supplement: Additional file 1: — Food frequency of participants and factor loadings for dietary patterns. (DOC 187 kb) [file 12889_2017_4819_MOESM1_ESM.doc]

Table 1 Food frequency of participants

|  |  | Total (N = 469) | | Breast cancer (N = 233) | | Control (N = 236) | |  |
| --- | --- | --- | --- | --- | --- | --- | --- | --- |
|  | Variable | Range | Mean + SD | Range | Mean + SD | Range | Mean + SD | *P* value |
| **Food frequency** | |  |  |  |  |  |  |  |
| Fish | | 0 – 3.00 | 0.22 + 0.38 | 0 – 3.00 | 0.29 + 0.48 | 0 – 1.00 | 0.15 + 0.24 | < 0.001 |
| Poultry (chicken, duck) | | 0 – 3.00 | 0.16 + 0.28 | 0 – 3.00 | 0.22 + 0.34 | 0 – 1.00 | 0.11 + 0.19 | < 0.001 |
| Livestock (pork, lean meat) | | 0 – 3.00 | 0.24 + 0.41 | 0 – 3.00 | 0.35 + 0.50 | 0 – 1.00 | 0.13 + 0.24 | < 0.001 |
| Streaked meat (fatty meat, ground meat) | | 0 – 2.00 | 0.06 + 0.17 | 0 – 2.00 | 0.09 + 0.22 | 0 – 1.00 | 0.03 + 0.10 | < 0.001 |
| Processed meat (sausage, pork  floss) | | 0 – 1.00 | 0.04 + 0.11 | 0 – 1.00 | 0.06 + 0.15 | 0 - 0.43 | 0.02 + 0.06 | < 0.001 |
| Organ meat (liver, heart, bowel) | | 0 – 1.00 | 0.02 + 0.08 | 0 – 1.00 | 0.03 + 0.09 | 0 - 0.43 | 0.02 + 0.06 | 0.108 |
| Shellfish (oyster clam) | | 0 – 1.00 | 0.05 + 0.11 | 0 – 1.00 | 0.06 + 0.13 | 0 - 0.57 | 0.03 + 0.07 | 0.003 |
| Seafood (shrimp, crab, squid) | | 0 – 1.00 | 0.05 + 0.10 | 0 – 1.00 | 0.06 + 0.12 | 0 - 0.43 | 0.03 + 0.07 | 0.002 |
| Soybeans (dried tofu) | | 0 – 3.00 | 0.46 + 0.57 | 0 – 3.00 | 0.47 + 0.65 | 0 – 3.00 | 0.44 + 0.47 | 0.485 |
| Soybeans (soybean milk) | | 0 – 3.00 | 0.41 + 0.46 | 0 – 3.00 | 0.40 + 0.49 | 0 – 3.00 | 0.42 + 0.42 | 0.732 |
| Milk (whole milk) | | 0 – 3.00 | 0.11 + 0.31 | 0 – 3.00 | 0.14 + 0.39 | 0 – 1.00 | 0.08 + 0.21 | 0.026 |
| Milk (low-fat milk) | | 0 – 2.00 | 0.11 + 0.29 | 0 – 2.00 | 0.13 + 0.34 | 0 – 1.00 | 0.09 + 0.23 | 0.124 |
| Milk (skim milk) | | 0 – 2.00 | 0.03 + 0.16 | 0 – 1.00 | 0.02 + 0.12 | 0 – 2.00 | 0.04 + 0.19 | 0.356 |
| Eggs | | 0 – 3.00 | 0.41 + 0.38 | 0 – 3.00 | 0.43 + 0.39 | 0 – 3.00 | 0.40 + 0.38 | 0.416 |
| Vegetables | | 0 – 3.00 | 1.90 + 0.71 | 0 – 3.00 | 1.89 + 0.82 | 0.13 – 3.0 | 1.91 + 0.57 | 0.737 |
| Mushrooms | | 0 – 3.00 | 0.46 + 0.54 | 0 – 3.00 | 0.43 + 0.59 | 0 – 3.00 | 0.48 + 0.48 | 0.233 |
| Fruit | | 0 – 3.00 | 1.15 + 0.79 | 0 – 3.00 | 1.15 + 0.87 | 0 – 3.00 | 1.15 + 0.70 | 0.948 |
| Fresh fruit juice | | 0 – 1.00 | 0.10 + 0.25 | 0 – 1.00 | 0.10 + 0.23 | 0 – 1.00 | 0.10 + 0.26 | 0.983 |
| Fried food | | 0 – 1.00 | 0.10 + 0.15 | 0 – 1.00 | 0.11 + 0.17 | 0 – 1.00 | 0.08 + 0.13 | 0.112 |
| Coffee | | 0 – 3.00 | 0.39 + 0.52 | 0 – 3.00 | 0.39 + 0.53 | 0 – 2.00 | 0.39 + 0.51 | 0.990 |
| Tea (black tea/green tea) | | 0 – 5.00 | 0.40 + 0.75 | 0 – 5.00 | 0.46 + 0.89 | 0 – 5.00 | 0.35 + 0.57 | 0.114 |
| Sweets (desserts, cake) | | 0 – 3.00 | 0.21 + 0.33 | 0 – 3.00 | 0.24 + 0.38 | 0 – 1.00 | 0.17 + 0.28 | 0.025 |
| Beverage | | 0 – 3.00 | 0.07 + 0.23 | 0 – 3.00 | 0.08 + 0.26 | 0 – 1.00 | 0.06 + 0.19 | 0.326 |
| Sugar (honey, candy) | | 0 – 1.00 | 0.11 + 0.27 | 0 – 1.00 | 0.08 + 0.23 | 0 – 1.00 | 0.15 + 0.31 | 0.004 |
| Sashimi | | 0 - 0.29 | 0.01 + 0.03 | 0 - 0.29 | 0.01 + 0.03 | 0 - 0.29 | 0.01 + 0.03 | 0.999 |
| Smoked meat (smoked bacon) | | 0 – 1.00 | 0.02 + 0.08 | 0 – 1.00 | 0.03 + 0.11 | 0 - 0.29 | 0.01 + 0.02 | 0.006 |
| Pickled food (kimchi, radish) | | 0 – 2.00 | 0.10 + 0.20 | 0 – 2.00 | 0.11 + 0.22 | 0 – 2.00 | 0.09 + 0.18 | 0.357 |
| Fermented food (miso) | | 0 – 1.00 | 0.08 + 0.15 | 0 – 1.00 | 0.08 + 0.16 | 0 – 1.00 | 0.09 + 0.15 | 0.444 |

| Table 2 All factor loadings (non-rotated) for the five major dietary patterns among participants | | | | | |
| --- | --- | --- | --- | --- | --- |
|  | Dietary pattern | | | | |
|  | Animal source | | Plant source | | |
| Food item | Meat | Processed meat | Fruit/Vegetables/Soybean | Dessert/sugar | Fermented food |
| Poultry (chicken, duck, goose) | 0.659 | -0.088 | 0.330 | -0.172 | -0.058 |
| Fish (sea fish, fresh water fish) | 0.492 | -0.033 | 0.568 | -0.162 | 0.061 |
| Livestock (pork, beef, lamb) | 0.636 | -0.055 | 0.259 | -0.242 | 0.114 |
| Seafood (shrimp, crab, squid, fish viscera, spawn) | 0.674 | 0.007 | 0.116 | 0.245 | -0.106 |
| Streaked meat (fatty meat) | 0.459 | -0.017 | 0.263 | 0.076 | 0.012 |
| Sashimi (sliced fresh fish meat) | 0.477 | -0.093 | 0.115 | -0.087 | 0.293 |
| Smoked meat (smoked chicken, smoked pork, smoked sausage, smoked bacon, smoked hot dog) | 0.434 | 0.136 | -0.341 | 0.006 | -0.381 |
| Fried food | 0.374 | 0.268 | -0.442 | -0.143 | -0.134 |
| Internal organs (liver, heart, kidney, colon, intestine) | 0.641 | 0.084 | -0.095 | 0.363 | -0.277 |
| Processed meat (sausage, bacon, cured meat, ham, pork floss) | 0.573 | -0.026 | -0.210 | 0.322 | -0.247 |
| Soybeans (dried tofu) | -0.188 | 0.706 | 0.114 | -0.026 | -0.101 |
| Soybeans (soybean milk) | -0.161 | 0.630 | 0.014 | -0.055 | -0.178 |
| Mushrooms | -0.108 | 0.634 | 0.106 | -0.114 | -0.020 |
| Fruit | -0.089 | 0.367 | 0.518 | 0.001 | -0.089 |
| Vegetables | -0.165 | 0.236 | 0.482 | 0.147 | -0.047 |
| Sugar (e.g., candy) | 0.109 | 0.225 | -0.181 | -0.063 | 0.600 |
| Sweets (e.g., cake, sweet bun) | 0.240 | 0.197 | -0.178 | -0.284 | 0.430 |
| Beverage | 0.381 | 0.181 | -0.352 | -0.210 | 0.165 |
| Fermented food (e.g., miso) | 0.093 | 0.281 | 0.027 | 0.648 | 0.319 |
| Pickled food (e.g., kimchi) | 0.100 | 0.148 | -0.064 | 0.588 | 0.353 |
| Eggs | 0.321 | 0.287 | -0.058 | -0.279 | -0.041 |
| Coffee | 0.232 | 0.003 | -0.141 | -0.157 | 0.136 |
| Milk (whole fat) | 0.245 | 0.127 | -0.010 | -0.083 | 0.024 |
| Milk (low fat) | 0.013 | -0.085 | 0.261 | -0.040 | 0.073 |
| Milk (skim fat) | -0.018 | -0.048 | 0.073 | -0.056 | -0.264 |
| Fresh fruit juice | 0.019 | 0.148 | -0.187 | -0.103 | -0.125 |
| Tea (e.g., black tea, green tea) | 0.189 | 0.133 | -0.027 | 0.015 | 0.035 |
| Eigenvalue | 3.636 | 1.977 | 1.810 | 1.500 | 1.383 |
| Variance explained (%) | 13.47 | 7.32 | 6.70 | 5.56 | 5.12 |

| Table 3 All factor loadings (rotated) for the five major dietary patterns among participants | | | | | |
| --- | --- | --- | --- | --- | --- |
|  | Dietary pattern | | | | |
|  | Animal source | | Plant source | | |
| Food item | Meat | Processed meat | Fruit/Vegetables/Soybean | Dessert/sugar | Fermented food |
| Poultry (chicken, duck, goose) | 0.744 | 0.100 | -0.066 | 0.041 | -0.123 |
| Fish (sea fish, fresh water fish) | 0.741 | -0.183 | 0.074 | 0.020 | -0.083 |
| Livestock (pork, beef, lamb) | 0.688 | 0.054 | -0.071 | 0.230 | -0.114 |
| Seafood (shrimp, crab, squid, fish viscera, spawn) | 0.594 | 0.327 | -0.088 | -0.070 | 0.257 |
| Streaked meat (fatty meat) | 0.519 | 0.047 | -0.023 | -0.014 | 0.118 |
| Sashimi (sliced fresh fish meat) | 0.464 | -0.016 | -0.165 | 0.308 | 0.070 |
| Smoked meat (smoked chicken, smoked pork, smoked sausage, smoked bacon, smoked hot dog) | 0.149 | 0.664 | -0.012 | -0.045 | -0.055 |
| Fried food | 0.045 | 0.604 | 0.068 | 0.261 | -0.072 |
| Internal organs (liver, heart, kidney, colon, intestine) | 0.433 | 0.564 | -0.070 | -0.166 | 0.313 |
| Processed meat (sausage, bacon, cured meat, ham, pork floss) | 0.321 | 0.559 | -0.195 | -0.127 | 0.262 |
| Soybeans (dried tofu) | -0.123 | 0.067 | 0.731 | 0.019 | 0.051 |
| Soybeans (soybean milk) | -0.152 | 0.162 | 0.639 | -0.005 | -0.015 |
| Mushrooms | -0.052 | 0.047 | 0.647 | 0.123 | -0.001 |
| Fruit | 0.207 | -0.246 | 0.531 | -0.182 | 0.016 |
| Vegetables | 0.120 | -0.305 | 0.392 | -0.248 | 0.129 |
| Sugar (e.g., candy) | -0.025 | -0.066 | 0.049 | 0.630 | 0.234 |
| Sweets (e.g., cake, sweet bun) | 0.102 | 0.057 | 0.048 | 0.614 | -0.019 |
| Beverage | 0.112 | 0.373 | -0.019 | 0.469 | -0.037 |
| Fermented food (e.g., miso) | 0.024 | -0.009 | 0.135 | 0.054 | 0.767 |
| Pickled food (e.g., kimchi) | -0.012 | -0.001 | -0.016 | 0.106 | 0.703 |
| Eggs | 0.234 | 0.274 | 0.220 | 0.247 | -0.171 |
| Coffee | 0.119 | 0.130 | -0.087 | 0.273 | -0.057 |
| Milk (whole fat) | 0.193 | 0.144 | 0.074 | 0.143 | -0.012 |
| Milk (low fat) | 0.169 | -0.228 | -0.002 | -0.036 | -0.028 |
| Milk (skim fat) | 0.036 | 0.056 | 0.026 | -0.221 | -0.164 |
| Fresh fruit juice | -0.092 | 0.226 | 0.099 | 0.052 | -0.105 |
| Tea (e.g., black tea, green tea) | 0.130 | 0.130 | 0.072 | 0.105 | 0.076 |
| Eigenvalue | 3.010 | 2.161 | 1.996 | 1.607 | 1.531 |
| Variance explained (%) | 11.15 | 8.01 | 7.39 | 5.95 | 5.67 |

| Table 4 Comparison of dietary oil consumption between the first and fourth quartiles of the meat/fat dietary pattern in breast cancer patients* | | | | | |
| --- | --- | --- | --- | --- | --- |
| Cooking or eating habit |  | | Q1 | Q4 |  |
|  |  | | N= 117 | N = 116 | *P* value |
| Sautéed meat in oil |  | | 0.23 + 0.87 | 2.79 + 1.55 | < 0.001 |
| Fried meat in oil |  | | 0.21 + 0.79 | 2.78 + 1.62 | < 0.001 |
| Sautéed or fried fish in oil |  | | 0.26 + 0.90 | 2.90 + 1.13 | < 0.001 |
| Sautéed vegetables in oil |  | | 0.17 + 0.67 | 1.79 + 0.83 | < 0.001 |
| Fried vegetables in oil |  | | 0.38 + 1.15 | 3.39 + 0.98 | < 0.001 |
| Eats poultry (chicken) with fat and skin | | 3.24 + 0.97 | | 3.67 + 0.80 | < 0.001 |
| Eats livestock (pork) with fat and skin | | 1.09 + 0.63 | | 1.11 + 0.57 | 0.819 |
| Eats rice with lard or gravy |  | | 0.62 + 1.10 | 1.60 + 1.15 | < 0.001 |
| Eats rice or noodles with sauce | | 1.53 + 1.06 | | 1.41 + 0.98 | 0.352 |
| Eats fried soy products |  | | 1.82 + 0.97 | 1.50 + 0.85 | 0.008 |
| Eats extra salt or seasoning |  | | 1.70 + 1.09 | 1.78 + 0.96 | 0.535 |
| Eats pickled or fermented foods with meal | | 1.76 + 0.96 | | 1.81 + 0.94 | 0.691 |
| * The analysis was carried out using independent two-sample t test. The range of scores is 1-6; higher score indicates higher frequency. | | | | | |
